# RESTORE: an exploratory trial of an online intervention to enhance self-efficacy to manage problems associated with cancer-related fatigue following primary cancer treatment: study protocol for a randomized controlled trial

**DOI:** 10.1186/1745-6215-14-184

**Published:** 2013-06-21

**Authors:** Chloe Grimmett, Jo Armes, Matthew Breckons, Lynn Calman, Jessica Corner, Deborah Fenlon, Claire Hulme, Christine M May, Carl R May, Emma Ream, Alison Richardson, Peter W F Smith, Lucy Yardley, Claire Foster

**Affiliations:** 1 Macmillan Survivorship Research Group, Faculty of Health Sciences, University of Southampton, Highfield Campus, Southampton, SO17 1BJ, UK; 2 Faculty of Health Sciences, University of Southampton, Highfield Campus, Southampton, SO17 1BJ, UK; 3 School of Psychology, University of Southampton, Highfield Campus, Southampton, SO17 1BJ, UK; 4 Florence Nightingale School of Nursing and Midwifery, King’s College London, James Clerk Maxwell Building, London, SE1 8WA, UK; 5 Institute of Health & Society, Newcastle University, Baddiley-Clark Building, Richardson Road, Newcastle upon Tyne, NE2 4AX, UK; 6 Leeds Institute of Health Sciences, Charles Thackrah Building, University of Leeds, 101 Clarendon Road, Leeds, LS2 9LJ, UK; 7 Southampton Statistical Sciences Research Institute, University of Southampton, Southampton, SO17 1BJ, UK

**Keywords:** Cancer survivors, Fatigue, Online, Self-efficacy, Self-management

## Abstract

**Background:**

There are over 25 million people worldwide living with or beyond cancer and this number is increasing. Cancer survivors face a range of problems following primary treatment. One of the most frequently reported and distressing symptoms experienced by cancer survivors is fatigue. There is growing support for survivors who are experiencing problems after cancer treatment to engage in supported self-management. To date there is some evidence of effective interventions to manage fatigue in this population; however, to our knowledge there are no online resources that draw on this information to support self-management of fatigue. This paper describes the protocol for an exploratory randomized controlled trial of an online intervention to support self-management of cancer-related fatigue after primary cancer treatment.

**Methods/design:**

This is a parallel-group two-armed (1:1) exploratory randomized controlled trial including 125 cancer survivors experiencing fatigue (scoring ≥4 on a unidimensional 11-point numeric rating scale for fatigue intensity) within five years of primary treatment completion with curative intent. Participants will be recruited from 13 NHS Trusts across the UK and randomized to either the online intervention (RESTORE), or a leaflet comparator (Macmillan Cancer Backup, *Coping with Fatigue*). The primary outcome is a change in Perceived Self-Efficacy for Fatigue Self-Management (as measured by the Perceived Self-Efficacy for Fatigue Self-Management Instrument). Secondary outcomes include impact on perception and experience of fatigue (measured by the Brief Fatigue Inventory), and quality of life (measured by the Functional Assessment of Cancer Therapy - General and the Personal Wellbeing Index). Outcome measures will be collected at baseline, 6 weeks (completion of intervention), and 3 months. Process evaluation (including telephone interviews with recruiting staff and participants) will determine acceptability of the intervention and trial processes.

**Discussion:**

Data from this trial will be used to refine the intervention and contribute to the design of an effectiveness trial. This intervention will be expanded to address other cancer-related problems important to cancer survivors following primary cancer treatment.

**Trial registration:**

ISRCTN67521059

## Background

There are around 25 million people worldwide living with or beyond cancer [1], and this number is increasing. With improvements in early diagnosis and treatments, patients are also living longer after cancer and cancer is beginning to be viewed as a long-term condition [2]. It is, therefore, of growing importance that the needs of this population are identified and effective interventions developed to resolve or support management of cancer-related problems.

The amount of evidence of the range and prevalence of problems faced by cancer survivors after completion of treatment is increasing and we know that cancer-related fatigue (CRF) is a frequently reported and distressing symptom [3,4], which can persist for a number of years after completion of treatments [5,6]. The exact prevalence of fatigue is unknown and appears to vary by tumour type, treatments received and time since diagnosis. However, it is generally accepted that around one-third of people will experience fatigue within the first year following treatment [7,8], and some studies report significantly higher rates [9,10].

Despite the frequency and debilitating effect of CRF, the evidence for pharmacological treatment is sparse [11]. A meta-analysis [12] and Cochrane review [13] suggest that physical activity may improve fatigue; however, the type, duration and intensity of the exercise that might be of benefit are not yet known. There is also some support for the use of cognitive behavioural therapy [14] and psychosocial support [15]; however, there has been a call for the development of further non-pharmacological interventions to help patients manage this symptom [11].

The importance of people taking an active role in managing long-term health conditions is increasingly recognized. It has been reported that this can increase a person’s self-efficacy, which is in turn associated with improved quality of life [16]. There is also evidence that self-care behaviours improve physical and psychological symptoms [17]. Our own research indicates that a key component of self-management for cancer survivors involves the rebuilding of confidence after cancer treatment [18], and that this confidence is amenable to change [19]. An emphasis on supported self-management strategies is important from the perspective of health service planners and policy makers because of the ever-increasing number of patients living with long-term consequences of cancer and associated economic costs. Indeed, in England, the National Cancer Survivorship Initiative have highlighted supported self-management as one of their key areas for development, and the National Coalition for Cancer Survivorship (United States) and the Australian Cancer Survivorship Centre have been established to promote research and development of services for people living with and beyond cancer.

There is mounting evidence for the efficacy of online interventions in the management of physical symptoms such as pain [20]. Online interventions have the advantage of being widely available to those with internet access (83% of the population of Great Britain [21]) and have been found to be cost-effective [22]. With an increasing proportion of older adults having access to the internet, there has also been an emergence of online interventions for this population. A recent review by Aalbers *et al*. [23] concluded that complex online interventions in the over 50s, designed to promote health behaviour change, were effective.

The development of RESTORE, an online intervention designed to increase self-efficacy to manage CRF, followed the recommendations of the Medical Research Council framework for developing and evaluating complex interventions [24]. It was informed by the conceptual framework of self-management after cancer treatment previously described by Foster *et al*. [25] and is underpinned by the principles of self-efficacy theory [26]. Bandura proposes that there are four sources of self-efficacy: performance accomplishment, verbal persuasion, vicarious experiences and physiological states. The RESTORE online intervention therefore includes components designed to enhance self-efficacy through these sources. The development of RESTORE is described elsewhere [27].

This paper describes the protocol of the RESTORE exploratory randomized controlled trial (RCT) and is written following CONSORT guidelines [28] and informed by SPIRIT guidance [29] for clinical trials. The aim of this study is to test the value (proof of concept) of this intervention and determine whether its use increases self-efficacy to self-manage CRF following completion of primary cancer treatment.

## Methods

### Design

This is an exploratory, parallel-group RCT and has been registered with the International Standard Randomized Controlled Trial Number Register (ISRCTN67521059). Participants will be randomized in a 1:1 ratio to receive either the RESTORE online intervention, or a leaflet comparator developed by Macmillan Cancer Backup, *Coping with Fatigue*[30]. Participants are randomized in blocks of four, clustered by NHS Trust. An independent statistician wrote a program using the statistical software R to generate a sequence of numbers for each NHS Trust. It was not possible to blind the coordinating research team to group allocation and the nature of the trial is such that blinding to patients could not be achieved. Data analysts will be blinded to group allocation when examining differences between groups.

This study has received ethical approval from the NRES Committee South Central – Oxford A, and research governance approval from the University Hospital Southampton NHS Foundation Trust, REF RHM CAN0875. This study has also been adopted by the UK Cancer Research Network Portfolio (ID 12769), which provides network resources, including Research Nurse and Clinical Trial Officer support for recruitment.

### Setting and participants

Participants will be recruited from 13 NHS Trusts across the UK (including sites in England, Scotland, Wales and Northern Ireland). We aim to recruit 125 patients who have completed primary treatment with curative intent for non-metastatic cancer within the last 5 years. Recruitment will take place between September 2012 and June 2013.

Participants are eligible for the study if they (1) are over 18 years of age, (2) are experiencing fatigue defined as scoring ≥4 on a unidimensional 11-point numeric rate scale for fatigue as suggested by the National Comprehensive Cancer Network [31], (3) are able to complete written records in English, (4) have or are willing to create an email account and have access to the internet, (5) have completed primary treatment for invasive cancer (patients are eligible for the study if they are receiving monoclonal antibodies or endocrine treatment as maintenance therapy). Patients will be excluded if (1) in the opinion of a relevant clinician they are unable to give informed consent, (2) have a previously diagnosed mental health condition that is likely to be exacerbated by participation in the intervention, (3) are too ill to engage in the intervention (determined by a member of the patient’s direct clinical care team).

Patients will be referred by their direct care team at an end-of-treatment appointment or during follow-up clinics to the hospital research team (for example, research nurses and clinical trial officers), who will screen patients for inclusion in the study. Screening will involve a face-to-face discussion with a member of the team. The study will be explained and patient status documented. Possible outcomes are (1) eligible and willing to participate, (2) eligible and unwilling to participate, and (3) ineligible. Wherever possible, the reason for declining participation and ineligibility will be recorded.

**Figure 1:**
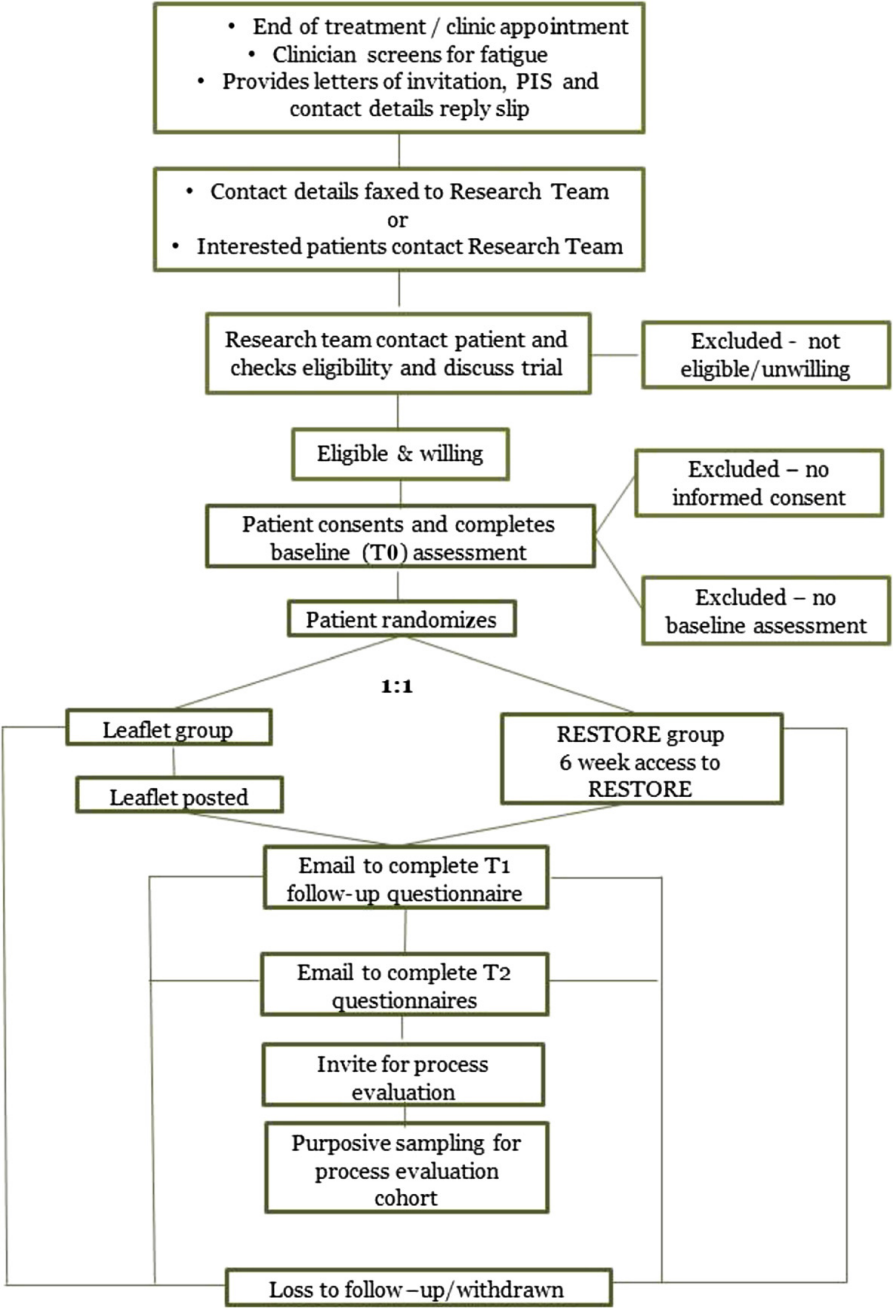
Flow of patients through the trial.

Eligible and willing patients will be given a letter of invitation, patient information sheet, reply slip and Freepost envelope. Patients who, after reading the documentation, are happy to take part will be asked to complete the reply slip and return it to the coordinating research team at the University of Southampton. A member of the coordinating research team will then contact the patient and discuss the study, answering any questions. Patients will then be sent an email including the website address for the RESTORE intervention and a unique study ID number. Patients register with their email, chosen unique password and study ID number and complete the online consent form and baseline assessment. A member of the coordinating research team then determines group allocation from the randomized list and an email is sent to participants notifying them of their group allocation. See Figure 1 for patient flow through the trial.

### The intervention

The RESTORE online intervention was developed using LifeGuide, open source software [32].

The purpose of this intervention is to increase participant’s self-efficacy to manage CRF. Table 1 summarizes the intervention and the components’ association with self-efficacy theory. Briefly, patients have six weeks to complete the five sessions of RESTORE. Patients are presented with sessions one and two (which are compulsory) at weekly intervals and can then choose from sessions three to five for the following three weeks. For example, a participant may choose to visit the same session three times, or visit each session once. It is expected (based on development work) that each session will take approximately 30 minutes to complete, depending on the amount of time spent engaging in the suggested activities.

**Table 1:** Intervention content and association with self-efficacy theory

**RESTORE sessions**	**Content**	**Mandatory/not mandatory**	**Self-efficacy theory construct**
Session 1: introduction	Defines CRF, possible causes and effects, and outlines purpose of the intervention	Mandatory	Not applicable
Session 2: goal setting	Introduces the concept of goal setting and planning; ‘SMART’ (specific, measurable, attainable, relevant, time-bound) goals are described	Mandatory	Performance accomplishments and verbal persuasion
Session 3: diet, sleep, exercise, home life and work	Describes how CRF may impact on these aspects of everyday life and how effective goal setting can help manage this interference	Not mandatory	Performance accomplishments and verbal persuasion
Session 4: thoughts and feelings	Psychological aspects of CRF and how these can be managed, including through goal setting	Not mandatory	Performance accomplishments and verbal persuasion
Session 5: talking to others	Describes the difficulties of talking to others (friends, family, colleagues, health professionals), and some strategies on how to manage this, including through goal setting	Not mandatory	Performance accomplishments and verbal persuasion
**Activities suggested throughout**			
Patient stories	Extracts from people affected by cancer and who have experienced fatigue; sharing their experiences and methods of self-management, written text and video clips	Not mandatory	Vicarious experiences
Self-monitoring	The use of a fatigue diary to monitor fatigue and understand its pattern	Not mandatory	Physiological experiences
	Monitoring fatigue each time a participant logs into RESTORE	Mandatory	
Feedback	Personal feedback on success of goal setting, planning and fatigue level	Mandatory	Performance accomplishments and verbal persuasion
Web links	Web links to useful resources	Not mandatory	Not applicable
Take a break buttons	Provided throughout to allow participants to rest during sessions if required	Not mandatory	Not applicable

The Macmillan Cancer Backup leaflet*, Coping with Fatigue* informed the content of the RESTORE sessions. Information and components of the intervention were also informed by the available evidence of fatigue management in cancer survivors. For example participants are encouraged to monitor activity patterns and engage in regular physical activity [12,13]. In addition, session four (managing thoughts and feelings) draws on the principles of cognitive behavioural therapy [14]. An expert design team supported the development of the RESTORE sessions.

Activities are available throughout RESTORE. These include a fatigue diary that can be downloaded from the website as a means of monitoring fatigue, in addition to completing a single-item assessment of fatigue during each session. Patient stories (as written text and video clips) are used throughout to provide examples of how people affected by CRF have managed the challenges faced. Automated tailored feedback based on goal-related progress and change in fatigue levels from the previous week are included throughout and there are links to useful resources such as mindfulness and relaxation training, and where to access information regarding financial support. ‘Take a break’ buttons are available during each session, allowing participants to rest if necessary.

During the trial, patients will receive automated emails encouraging them to complete the next session if they have not done so within seven days. Similar emails will be sent to prompt completion of outcomes measures.

### Comparator group

The leaflet *Coping with Fatigue* produced by Macmillan Cancer Backup will be sent to those in the comparator group. This information leaflet on managing CRF is widely available in the UK through local information services and used by clinical teams. Participants in the comparator group will be given access to the RESTORE online intervention once all outcomes measures have been completed.

### Assessments

During screening, the participant’s date of birth, sex and cancer type will be recorded to allow analysis of factors influencing uptake.

Baseline assessment (T0) will be completed online immediately after participants consent to the study and prior to randomization. Demographic information will include age, sex, ethnicity, marital status, educational status, accommodation type and postcode. Clinical information will also be abstracted from patients’ clinical records by a member of their hospital research team. This will include information on cancer diagnosis (date, type and stage) and treatments received.

The primary outcome measure is the Perceived Self-Efficacy for Fatigue Self-Management Instrument (PSEFSM) [33]. The instrument includes six items and respondents are asked to report their degree of self-efficacy in performing various tasks on an eleven-point scale. This measure has been shown to have good reliability (Cronbach’s coefficient αof 0.92), construct validity and generalizability [33].

A number of secondary outcome measures will be also assessed.

Self-efficacy for managing chronic disease will be measured by the Self-Efficacy for Managing Chronic Disease 6-item Scale [34]. This scale has response options from 1 to 10 (with 10 being most confident) for six items. Respondents are asked to rate their confidence in performing various activities. The scale has good internal validity and has been used in cancer populations [35].

Quality of life will be measured by the Functional Assessment of Cancer Therapy - General (FACT-G) [36]. This is a 27-item questionnaire consisting of four subscales: physical wellbeing (seven items), social wellbeing (seven items), emotional wellbeing (six items) and functional wellbeing (seven items). Respondents indicate how true each statement was for them during the previous seven days on a five-point Likert scale. Higher scores indicate higher quality of life. This measure has proven validity and reliability [36]. The Personal Wellbeing Index (PWI) will also be used to measure quality of life. This index includes seven items, which deconstruct the global question, ‘How satisfied are you with your life as a whole?’ and has been found to have good reliability (Cronbach’s α 0.70 to 0.85) [37]. Response options are 0 to 10, with higher scores denoting higher satisfaction with life.

Depression will be assessed using the Patient Health Questionnaire (PHQ-9). This is a nine-item scale consisting of two components; one assesses symptoms and functional impairment, the other assesses severity. This tool is based on the diagnostic criteria for depressive disorder. This is a reliable and valid measure of depression [38] and has been used extensively, including among cancer populations [39].

Fatigue will be assessed using the Brief Fatigue Inventory. This is a nine-item scale. Items one to three assess current, usual and worst levels of fatigue (during the previous 24 hours). Items four to nine examine the extent to which fatigue interferes with everyday life, such as mood, relationships and enjoyment. This scale has been used in samples of cancer survivors and has been shown to have good validity and reliability (Cronbach’s α 0.95 to 0.96 [40].

For economic evaluation, items assessing health service use, caring responsibilities and improvements from a patient perspective will also be included. The objective of the economic analysis is to identify the costs (and cost savings) associated with RESTORE compared with the leaflet comparator. The analysis will take the perspective of the health and social care sectors, including third-sector organizations (for example, hospice care). Patients’ use of these resources will be collected by way of a bespoke patient questionnaire. The resources associated with provision and implementation of RESTORE will be included in the analysis. These will be based on routine data, such as administrative records, as well as a detailed description of the implementation and the development process of the intervention. Unit costs for health service resources will be obtained from national sources such as the Personal Social Services Research Unit (PSSRU), the British National Formulary and NHS Reference cost database. Where national unit costs are not available, the finance departments of trusts participating in the study will be asked to provide local cost data. The mean of these costs will be used as the unit cost estimate in the analysis. The results will be presented alongside the primary and secondary outcomes as a cost consequence analysis.

All primary and secondary outcome measures will be assessed at baseline (T0), six weeks (T1; completion of intervention) and three months (T2; end of study). T2 will act as our primary endpoint.

This is an exploratory trial and therefore a mixed-methods process evaluation will evaluate the feasibility and acceptability of the intervention. Quantitatively, adherence to the intervention (that is, number of sessions completed) will be examined, as will completion rates of questionnaire items at all three time points. Recruitment rates will be calculated based on the number of eligible patients approached versus those who consent to take part in the trial. The proportion of ineligible patients will also be described and reasons for ineligibility documented. Eligible patients who decline participation will also be asked to volunteer a reason for this. Acceptability of the intervention will be inferred by attrition rates. Additionally, semistructured telephone interviews with participants will explore feasibility and acceptability in more depth. We will recruit a maximum variation sample of men (*n* ≈ 15) and women (*n* ≈ 15) where variation will be by (a) cancer type and time since treatment completion, and (b) compliance with the intervention. The interview schedule will be structured to identify, describe and explain the impact of CRF on participants, the perceived acceptability of online interventions and the value of RESTORE to them (for example, improved confidence, reduced interference of CRF in daily life), and factors that promote or inhibit the integration of RESTORE into everyday life. Telephone interviews will be transcribed and analyzed using directed content analysis [41]. Analysis will be informed by Normalization Process Theory [42,43].

### Sample size

We aim to recruit 125 patients to this trial. Currently, there is no definitive guidance on the size of exploratory trials. We feel that this is an achievable sample size based on the resources available and is adequate to allow for a relatively high attrition rate while still leaving a reasonable final sample. Also, the primary outcome measure (PSEFSM) could not be used to determine a power calculation as the measure is new and insufficient data are available. A definitive sample size for a large scale RCT will be determined from results of this trial.

### Analysis

The principal analysis will involve fitting between group, repeated measures models with two levels of treatment variable (intervention group, leaflet group) and three time points (T0, T1, T2) to the primary and secondary outcome measures. Distribution of the outcomes will be inspected to identify the appropriate form of the response variables for the modelling. Model assumptions will be checked and outcomes will be transformed where there is strong evidence of non-normality, for example by taking logs, dichotomizing or categorizing into three or more ordered categories. The appropriate mixed effects, repeated measures model (normal, binary or ordinal) will be fitted with random coefficients on the time points and a random intercept for trust to control for within-subject and within-Trust correlations. All data will be analyzed blind to treatment condition and intention to treat analysis will be undertaken. Auxiliary analyses will assess the interaction of potential modifiers (for example, age, sex, socio-economic status, cancer treatment received, cancer type) with treatment in the models. Change over time of the primary and all secondary outcome measures will be examined.

### Study funding

This study is funded by Macmillan Cancer Support as part of a programme of research.

### Serious adverse events reporting and monitoring

It is not anticipated that there will be any risk to participants, therefore only serious adverse events will be reported. Any serious adverse events deemed to be related to the intervention or due to participation in the study will be reported to the chief investigator within 24 hours of the team learning of its occurrence.

## Discussion

The RESTORE resource provides a novel intervention and this trial will explore its feasibility as a self-management tool to support people living with CRF following primary cancer treatment. To the best of our knowledge this is the first online intervention designed to increase self-efficacy in managing CRF in patients who have completed primary cancer treatment. As the number of people living with and beyond cancer continues to increase, there will be a growing need for evidence-based support tools.

The results of this study will determine whether RESTORE is acceptable to people living with and beyond cancer, and whether recruitment into a full-scale trial is both warranted and feasible.

This study has strengths and weaknesses. We have only included patients with curative disease and who are within five years of treatment completion. The intervention is also only available in English. However, the exploratory nature of the study will permit refinement of the intervention and consideration of optimal study design for a larger trial. The study will also provide important information as to whether this typically older population of cancer survivors is accepting of an online based intervention, and if the intervention is feasible and acceptable. It is hoped that this intervention will be expanded in the future to support the management of other cancer-related problems.

## Trial status

Recruiting. Recruitment closes June 2013.

## Abbreviations

CRF: Cancer-related fatigue; FACT-G: Functional Assessment of Cancer Therapy - General; PHQ-9: Patient Health Questionnaire; PSEFSM: Perceived Self-Efficacy for Fatigue Self-Management Instrument; PSSRU: Personal Social Services Research Unit; PWI: Personal Wellbeing Index; RCT: Randomized controlled trial

## Competing interests

The authors declare that they have no competing interests.

## Authors’ contributions

CG contributed to the study design and conception and drafted the protocol and manuscript for the study. JA, ER and DF contributed to the study design and critical revisions of both the protocol and manuscript. MB and LC contributed to the study design and conception and critical revisions of both the protocol and manuscript. JC contributed to the conception of the study and critical revisions of the manuscript. CH contributed to critical revisions of the manuscript and was the key consultant for health economic evaluation. CMM contributed to the study design and drafting and critical revisions of both the protocol and manuscript. CRM, AR and LY contributed to the study design and conception and drafting and critical revisions of both the protocol and manuscript. PS contributed critical revisions of the manuscript and was the key statistician for the study, advising of power calculation and analytical plans. CF was the chief investigator and grant holder for the study and Macmillan Survivorship Research Group Programme, conceived of the study and contributed to the study design, conception, drafting and critical revision of the protocol and manuscript. All authors read and approved the final manuscript.

## References

[B1] Research WCRFAIfC Food, Nutrition, Physical Activity, and the Prevention of Cancer: A Global Perspective 2007 Washington DC

[B2] World Health Organization Health Topics: Chronic Diseases[http://www.who.int/topics/chronic_diseases/en/]

[B3] Richardson A Fatigue in cancer patients: a review of the literature Eur J Cancer Care 1995 4 20 32 10.1111/j.1365-2354.1995.tb00049.x 7620651

[B4] Schlairet M Heddon MA Griffis M Piloting a needs assessment to guide development of a survivorship program for a community cancer center Oncol Nurs Forum 2010 37 501 508 10.1188/10.ONF.501-508 20591810

[B5] Hjermstad MJ Fossa SD Oldervoll L Holte H Jacobsen AB Loge JH Fatigue in long-term Hodgkin’s disease survivors: a follow-up study J Clin Oncol 2005 23 6587 6595 10.1200/JCO.2005.09.936 16170166

[B6] Fossa SD Dahl AA Loge JH Fatigue, anxiety, and depression in long-term survivors of testicular cancer J Clin Oncol 2003 21 1249 1254 10.1200/JCO.2003.08.163 12663711

[B7] Bower JE Ganz PA Desmond KA Rowland JH Meyerowitz BE Belin TR Fatigue in breast cancer survivors: occurrence, correlates, and impact on quality of life J Clin Oncol 2000 18 743 10673515 10.1200/JCO.2000.18.4.743

[B8] Broeckel JA Jacobsen PB Horton J Balducci L Lyman GH Characteristics and correlates of fatigue after adjuvant chemotherapy for breast cancer J Clin Oncol 1998 16 1689 1696 9586880 10.1200/JCO.1998.16.5.1689

[B9] Curt GA Breitbart W Cella D Groopman JE Horning SJ Itri LM Johnson DH Miaskowski C Scherr SL Portenoy RK Vogelzang NJ Impact of cancer-related fatigue on the lives of patients: new findings from the fatigue coalition Oncologist 2000 5 353 360 10.1634/theoncologist.5-5-353 11040270

[B10] Langston B Armes J Levy A Tidey E Ream E The prevalence and severity of fatigue in men with prostate cancer: a systematic review of the literature Support Care Cancer 2013 21 1761 1771 10.1007/s00520-013-1751-5 23455492

[B11] Minton O Richardson A Sharpe M Hotopf M Stone P A systematic review and meta-analysis of the pharmacological treatment of cancer-related fatigue J Natl Cancer Inst 2008 100 1155 1166 10.1093/jnci/djn250 18695134

[B12] Jacobsen PB Donovan KA Vadaparampil ST Small BJ Systematic review and meta-analysis of psychological and activity-based interventions for cancer-related fatigue Heathl Psychol 2007 26 660 667 10.1037/0278-6133.26.6.660 PMC2398706 18020836

[B13] Cramp F Byron-Daniel J Exercise for the management of cancer-related fatigue in adults Cochrane Database Syst Rev 2012 10.1002/14651858.CD006145.pub3 PMC8480137 23152233

[B14] Armes J Chalder T Addington-Hall J Richardson A Hotopf M A randomized controlled trial to evaluate the effectiveness of a brief, behaviorally oriented intervention for cancer-related fatigue Cancer 2007 110 1385 1395 10.1002/cncr.22923 17661342

[B15] Reif K de Vries U Petermann F What does really help against cancer-related fatigue? An overview of systematic reviews Pflege 2012 25 439 457 10.1024/1012-5302/a000246 23188754

[B16] Lorig KR Ritter P Stewart AL Sobel DS Brown BW Bandura A Gonzalez VM Laurent DD Holman HR Chronic disease self-management program - 2-year health status and health care utilization outcomes Med Care 2001 39 1217 1223 10.1097/00005650-200111000-00008 11606875

[B17] Lev EL Bandura’s theory of self-efficacy: applications to oncology Sch Inq Nurs Pract 1997 11 21 37 Discussion, 39–43 9188268

[B18] Foster C Fenlon D Recovery and self-management support following primary cancer treatment Br J Cancer 2011 105 S21 S28 22048029 10.1038/bjc.2011.419 PMC3251956

[B19] Bandura A Social Foundations of Thought and Action: A Cognitive Social Theory 1986 New York: Prentice Hall, Englewood Cliffs

[B20] Buhrman M Faltenhag S Strom L Andersson G Controlled trial of internet-based treatment with telephone support for chronic back pain Pain 2004 111 368 377 10.1016/j.pain.2004.07.021 15363881

[B21] Office for National Statistics Internet Access - Households and Individuals, 2012 Part 2 2013 Newport, Wales

[B22] Cobiac LJ Vos T Barendregt JJ Cost-effectiveness of interventions to promote physical activity: a modelling study PLoS Med 2009 6 e1000110 10.1371/journal.pmed.1000110 19597537 PMC2700960

[B23] Aalbers T Baars MAE Rikkert MGMO Characteristics of effective internet-mediated interventions to change lifestyle in people aged 50 and older: a systematic review Ageing Res Rev 2011 10 487 497 10.1016/j.arr.2011.05.001 21628005

[B24] Medical Research Council Developing and Evaluating Complex Interventions: New Guidance 2008 London: MRC

[B25] Foster CL Roffe L Scott I Cotterell P Self Management of Problems Experienced Following Primary Cancer Treatment: An Exploratory Study 2010 London: Macmillan Cancer Support

[B26] Bandura A Self Efficacy: The Exercise of Control 1997 New York: WH Freerman and Company

[B27] Grimmett C Breckons M Calman L Corner J Fenlon D Richardson A Smith P Yardley L Foster C Team D RESTORE: a web-based intervention to support self-management of cancer related fatigue following primary cancer treatment [abstract] Psycho-Oncology 2013 22 10 10

[B28] Moher D Hopewell S Schulz KF Montori V Gotzsche PC Devereaux PJ Elbourne D Egger M Altman DG CONSORT 2010 explanation and elaboration: updated guidelines for reporting parallel group randomised trials Int J Surg 2012 10 28 55 10.1016/j.ijsu.2011.10.001 22036893

[B29] Chan AW Tetzlaff JM Gøtzsche PC Altman DG Mann H Berlin JA Dickersin K Hróbjartsson A Schulz KF Parulekar WR Krleza-Jeric K Laupacis A Moher D SPIRIT 2013 explanation and elaboration: guidance for protocols of clinical trials BMJ 2013 346 e7586 10.1136/bmj.e7586 23303884 PMC3541470

[B30] Macmillan Cancer Support Coping with Fatigue 2011 5 MAC11664 [http://be.macmillan.org.uk/be/p-284-coping-with-fatigue.aspx]

[B31] National Comprehensive Cancer Network NCCN Clinical Practice Guidelines in Oncology - Cancer-Related Fatigue 2013 Fort Washington PA Version 1.2013

[B32] Hare J Osmond A Yang Y Wills G Weal M De Roure D Joseph J Yardley L LifeGuide: a platform for performing web-based behavioural interventions WebSci’09: Society On-Line, 18–20 Mar 2009, Athens, Greece [http://eprints.soton.ac.uk/267201/]

[B33] Hoffman AJ von Eye A Gift AG Given BA Given CW Rothert M The development and testing of an instrument for perceived self-efficacy for fatigue self-management Cancer Nurs 2011 34 167 175 10.1097/NCC.0b013e31820f4ed1 21512344 PMC3312582

[B34] Lorig K Stewart A Ritter P Gonzalez V Laurent D Lynch J Outcome Measures for Health Education and Other Health Care Interventions 1996 London: SAGE Publications

[B35] Lorig K Brown B Ung E Chastain R Shoo RS Holman H Development and evaluation of a scale to measure the perceived self-efficacy of people with arthritis Arthritis Rheum 1989 32 37 44 10.1002/anr.1780320107 2912463

[B36] Cella DF Tulsky DS Gray G Sarafian B Linn E Bonomi A Silberman M Yellen SB Winicour P Brannon J The functional assessment of cancer therapy scale: development and validation of the general measure J Clin Oncol 1993 11 570 579 8445433 10.1200/JCO.1993.11.3.570

[B37] Cummins RA Eckersley R Pallant J Van Vugt J Misajon R Developing a national index of subjective wellbeing: the Australian unity wellbeing index Soc Indic Res 2003 64 159 190 10.1023/A:1024704320683

[B38] Kroenke K Spitzer RL Williams JBW The PHQ-9: validity of a brief depression severity measure J Gen Intern Med 2001 16 606 613 10.1046/j.1525-1497.2001.016009606.x 11556941 PMC1495268

[B39] Johns SA Kroenke K Krebs EE Theobald DE Wu J Tu W Longitudinal comparison of three depression measures in adult cancer patients J Pain Symptom Manag 2013 45 71 82 10.1016/j.jpainsymman.2011.12.284 PMC3538946 22921152

[B40] Mendoza TR Wang XS Cleeland CS Morrissey H Johnson BA Wendt JK Huber SL The rapid assessment of fatigue severity in cancer patients - use of the brief fatigue inventory Cancer 1999 85 1186 1196 10.1002/(SICI)1097-0142(19990301)85:5<1186::AID-CNCR24>3.0.CO;2-N 10091805

[B41] Hsieh HF Shannon SE Three approaches to qualitative content analysis Qual Heal Res 2005 15 1277 1288 10.1177/1049732305276687 16204405

[B42] May C Towards a general theory of implementation Implement Sci 2013 8 18 10.1186/1748-5908-8-18 23406398 PMC3602092

[B43] May C Agency and implementation: understanding the embedding of healthcare innovations in practice Soc Sci Med 2013 78 26 33 23246396 10.1016/j.socscimed.2012.11.021

